# Partners in death: a role for p73 and NF-kB in promoting apoptosis

**DOI:** 10.18632/aging.100033

**Published:** 2009-03-28

**Authors:** Karen H. Vousden

**Affiliations:** The Beatson Institute for Cancer Research, Garscube Estate, Glasgow, G61 1BD, UK

**Keywords:** p73, NF-kB, NOXA, genotoxic stress

The p53 and NF-kB families of transcription factors play key roles in the regulation of cell death and cell viability.  Best understood are the functions of p53 - and its related family member p73 - in driving cell death, and the ability of NF-kB to promote cell survival [1-3]. However, more recent reports have illustrated a deeper complexity to this paradigm and it is clear that under some circumstances p53 can promote cell survival, while NF-kB can participate in the activation of cell death [3-6]. A report by Martin et al. in this issue of *Aging* provides a further link in these pathways by showing that NF-kB has a critical pro-apoptotic role in p73-dependent death following DNA damage.

In their study, Martin et al. show that DNA damage-induced apoptosis in immortalised and transformed mouse embryo fibroblasts depends on the presence of the NF-kB subunit, p65. Cell devoid of p65 failed to die following treatment with etoposide, or exposure to ultraviolet light (UV), although sensitivity to apoptosis was regained in these cells following transduction of a retrovirus expressing p65. Previous studies had shown a similar dependence on p65 for the induction of p53-induced death in several systems [7]. However, in this case the cells used by Martin et al. expressed an inactive mutant form of p53 - so these studies reveal a further role for p65 in the activation of p53-independent apoptosis.

The authors sought to determine at what stage NF-kB was essential for cell death induction. They first analyzed for defects in the core apoptotic machinery, including analysis of expression of Apaf-1 and caspases 2, 3 and 9, but no differences were found.  In addition, analysis of the ability of cytochrome *c* to induce caspase activation in a cell free system derived from extracts of these p65-null cells indicated that the core apoptotic machinery was indeed functional in these cells.  In whole cells, however, it was clear that the loss of p65 resulted in a marked impairment in the ability to release cytochrome *c* from mitochondria following genotoxic stress, indicating that a defect was present in the p65-null cells upstream of this process.

Since p65 is a transactivating member of the NF-kB family, it was reasoned by the authors that there should be genes whose expression is impaired due to loss of p65, and that these may include genes required for cell death induction in these cells.  Microarray analysis comparing p65-null with p65-reconstituted MEFs revealed that the mRNA for *Noxa*, a BH3-only pro-apoptotic member of the Bcl-2 family, was considerably reduced in p65-null cells.  Interestingly, *Noxa* has previously been shown to be a target gene of p53 and a critical component of p53-mediated cell death in certain cellular contexts [8]. Moreover, since the release of cytochrome *c* from mitochondria is known to be regulated by members of the Bcl-2 family [9], Martin et al. decided to investigate this change in *Noxa* expression further. Other members of the BH3 protein family were not affected by p65, suggesting that the dependency on p65 for expression was exclusive to *Noxa*.  Induction of *Noxa* expression is known to occur following genotoxic stress [8,10,11], but the authors observed that this induction is also absent in p65-null cells.  But is this failure to induce *Noxa* important?  It would seem so, since the authors were able to show that expression of *Noxa* in the p65-null cells restored apoptotic sensitivity.

*Noxa* belongs to a group of genes that are transcriptionally activated by members of the p53 family, including p73, which are themselves activated in response to DNA damage. Since their cells did not contain functional p53, Martin et al. considered whether p73 might be playing a role in the regulation of *Noxa* expression. While the cells lacking p65 showed a rather weak activation of p73β expression in response to etoposide, both background and stress induced expression of p73β was greatly enhanced in p65-reconstituted cells. The importance of p73β in driving *Noxa* expression was established using a naturally occurring dominant-negative form of p73 (DN-p73β). Interestingly, although the induction of *Noxa* in the p65-reconstituted cells was completely ablated by DN-p73β, the ectopic expression of a transactivation-competent version of p73β failed to activate expression of *Noxa* in the p65-null cells. So taken together it would seem that both p65 and p73 are required for *Noxa* induction - both are necessary but neither is sufficient.

These findings not only provide another context in which NF-kB has a pro-apoptotic role, but also highlight an interesting interplay between NF-kB and p73. Clearly, however, a number of questions still remain to be addressed.  Most notably, what is the nature of the role of p65 in the induction of *Noxa*? While the levels of p73β are clearly lower in the absence of p65, the failure of overexpressed p73β to drive *Noxa* expression in these cells suggests that p65 is doing something beyond simply regulating p73β levels. One obvious possibility is that p65 contributes directly to the transcriptional activation of *Noxa*, and it would be of interest to determine whether the *Noxa* promoter contains an NF-kB binding site. Alternatively, does p65 control the expression of another transcription factor responsible for *Noxa* expression, or is the effect of p65 on *Noxa* completely independent of the *Noxa* promoter - for instance, through microRNA control?  In this regard, it would be interesting to know the phosphorylation status of p65 in these cells following DNA damage, since this has been shown to determine whether p65 functions as a transcriptional activator or repressor [12].  The potential role of other NF-kB family members also remains to be determined - including p50, the usual binding partner for p65, and the p52/RelB components of the non-canonical NF-kB pathway that has recently been shown to play a role in pro-apoptotic NF-kB signaling [13,14]. It might therefore be informative to determine the influence of p50 and p52/RelB on the activation of *Noxa* expression.

Although the study from Martin et al. focuses on the interaction of NF-kB with p73, it is tempting to speculate that aberrant *Noxa* regulation may also underlie the pro-apoptotic role of NF-kB in other settings.  An obvious question is whether NF-kB is required for the activation of *Noxa* by p53. Could this explain the defect in p53-mediated apoptosis seen following loss of p65 in some contexts? Since p73 and p53 are likely to operate through the same transcriptional control element, any cooperation between NF-kB and p73 is likely to extend to p53 as well. Although such a mechanism is an attractive model to explain some aspects of the interaction between p53 and NF-kB, this relationship is clearly much more complicated. Indeed, several studies have documented the ability of NF-kB to inhibit p53-mediated apoptosis, for example through an NF-kB-mediated decrease of p53 stability [15]. We still seem to be a long way from fully understanding the intricate dance between NF-kB and p53.

Finally, it is worth considering the implications of these findings on the development and treatment of human cancer.  Transactivating (TA)-p73 has recently been shown to be a tumour suppressor in its own right, and chemotherapeutic responses have been thought for some time to be mediated, at least in part, through induction of p73 [16,17]. NF-kB, on the other hand, is generally considered to play a tumor-promoting role, through its anti-apoptotic activity, and simultaneous activation of p53 and inhibition of NF-kB is likely to be a highly desirable goal for cancer therapeutics in many situations [18]. The pursuit of NF-kB inhibitors for cancer therapy will be tempered, however, by the paradoxical observations that inhibition of NF-kB can also contribute to tumor development in xenografts and mouse models of skin cancer, and that expression of β-catenin and HSCO has been shown to promote oncogenesis - at least in part - by inhibiting NF-kB [5,6,19-21]. We obviously need to understand this Jekyll and Hyde behavior of NF-kB to make the best use of any drugs based on inhibiting its activity. To this end, the findings presented by Martin et al. provide an interesting mechanistic basis to explain at least some of the pro-apoptotic functions of NF-kB.
